# 4,12-Bis(2,2-dibromo­vinyl)[2.2]paracyclo­phane

**DOI:** 10.1107/S1600536809002475

**Published:** 2009-02-13

**Authors:** Sébastien Clément, Laurent Guyard, Michael Knorr, Christian Däschlein, Carsten Strohmann

**Affiliations:** a Institut UTINAM UMR CNRS 6213, Université de Franche-Comté, 16 Route de Gray, La Bouloie, 25030 Besançon, France; b Technische Universität Dortmund, Anorganische Chemie Otto-Hahn-Strasse 6, D-44227 Dortmund, Germany

## Abstract

In the title compound, C_20_H_16_Br_4_, both vinylic substituents were introduced by a Corey–Fuchs reaction using 4,12-diform­yl[2.2]paracyclo­phane as starting material. The title compound may be used as a valuable precursor for the synthesis of diethyn­yl[2.2]paracyclo­phane. The title mol­ecule is centrosymmetric with a crystallographic center of inversion between the centers of the two phenyl rings. A strong tilting is observed with an inter­planar angle between the best aromatic plane and the vinyl plane of 49.4 (5)°. No significant inter­molecular inter­actions are found in the crystal.

## Related literature

For related structures of halovinyl compounds, see: Clément *et al.* (2007*a* ▶,*b* ▶); Jones *et al.* (1993 ▶); Hua *et al.* (2006 ▶). For ethynyl-functionalized[2.2]paracyclo­phanes, see: Jones *et al.* (2007 ▶). For the Corey–Fuchs reaction, see: Corey *et al.* (1972 ▶). For applications of [2.2]paracyclo­phanes, see: Hopf *et al.* (2008 ▶). For an alternative to the classical Sonogashira synthesis, see: Morisaki *et al.* (2003 ▶).
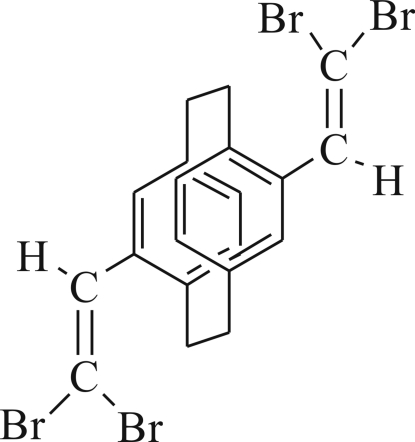


## Experimental

### 

#### Crystal data

C_20_H_16_Br_4_
*M* *_r_* = 575.97
Orthorhombic, 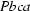

*a* = 12.155 (1) Å
*b* = 8.3819 (9) Å
*c* = 18.335 (2) Å
*V* = 1867.9 (3) Å^3^
*Z* = 4
Mo *K*α radiation
μ = 8.62 mm^−1^
*T* = 173 (2) K
0.30 × 0.20 × 0.10 mm

#### Data collection

Bruker APEX CCD diffractometer
Absorption correction: multi-scan (*SADABS*; Bruker, 1999 ▶) *T*_min_ = 0.141, *T*_max_ = 0.418
16030 measured reflections
2043 independent reflections
1766 reflections with *I* > 2σ(*I*)
*R*_int_ = 0.045

#### Refinement

*R*[*F* ^2^ > 2σ(*F* ^2^)] = 0.032
*wR*(*F* ^2^) = 0.073
*S* = 1.08
2043 reflections
109 parameters
H-atom parameters constrained
Δρ_max_ = 0.84 e Å^−3^
Δρ_min_ = −0.60 e Å^−3^

### 

Data collection: *SMART* (Bruker, 2001 ▶); cell refinement: *SAINT-Plus* (Bruker, 2001 ▶); data reduction: *SAINT-Plus*; program(s) used to solve structure: *SHELXS97* (Sheldrick, 2008 ▶); program(s) used to refine structure: *SHELXL97* (Sheldrick, 2008 ▶); molecular graphics: *ORTEP-3* (Farrugia, 1997 ▶); software used to prepare material for publication: *SHELXL97*.

## Supplementary Material

Crystal structure: contains datablocks global, I. DOI: 10.1107/S1600536809002475/im2094sup1.cif

Structure factors: contains datablocks I. DOI: 10.1107/S1600536809002475/im2094Isup2.hkl

Additional supplementary materials:  crystallographic information; 3D view; checkCIF report

## Figures and Tables

**Fig. 1.**
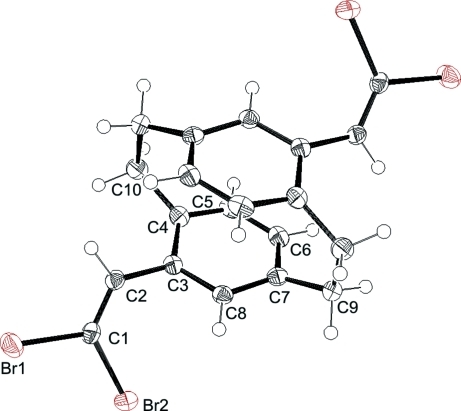
ORTEP plotof 2b with 30% probability level.

**Fig. 2.**
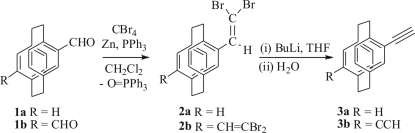
The formation of the title compound.

## supplementary crystallographic information

### Comment

In the context of our research in developing novel π-conjugated functionalized [2.2]paracyclophanes and ferrocenes (Clément *et al.*, 2007*a*) for potential applications in coordination chemistry, we have recently reported an alternative to the classical Sonogashira synthesis (Morisaki *et al.*, 2003) for the synthesis of ethynyl functionalized [2.2]paracyclophanes (Scheme 2). Our route involves a Corey-Fuchs reaction (Clément *et al.*, 2007*b*) and subsequent dehydobromation induced by a strong base. In the first step, an ylide species, formed *in situ* by the interaction of zinc dust, CBr_4_ and PPh_3_, reacts with the formyl derivative 1a or 1b leading to the dibromoolefin derivatives 2a or 2b. The molecular structure of the vinylic intermediate 2b was elucidated by an single-crystal X-ray diffraction study (Figure 1).

2 b possesses a crystallographic center of inversion in the middle of the cyclophane framework. Bond lengths and angles may be considered as normal. Distortions typical of [2.2]paracyclophane systems, *e.g.* lengthened C—C bonds and widened angles in the bridges, narrowed ring bond angles at the bridgehead atoms, and boat-like distortion of the rings (the bridgehead atoms lie significantly out of the plane of the other four ring atoms) are observed. As previously noticed for the monodibromoolefin compound 2a, the alkenyl unit of 2b is strongly tilted. The two best planes of the arene (C3, C4, C5, C6, C7, C8; plane 1) and the vinyl-group (Br1, Br2, C1, C2; plane 2) possess a cutting angle of the normals of 49.4 (5)°

Contrary to a recently published, related system, no significant intermolecular interactions are observed for 2b due to inproper orientation of the molecules relative to each other (Hopf *et al.*, 2007).

In the light of our recent work on the reactivity of (2,2-dibromovinyl)ferrocene towards thiolates (Clément *et al.*, 2007*b*), the vinylic intermediates 2a and 2b should be promising starting materials for building new ligand systems. Synthesis, reactivity and photochemical properties of these new compounds will be reported in due course.

### Experimental

PPh_3_ (4.20 g, 16.0 mmol), CBr_4_ (5.31 g, 16.0 mmol) and zinc dust (1.05 g, 16.0 mmol) are placed in a Schlenk tube and CH_2_Cl_2_ (45 ml) is added slowly. The mixture is stirred at room temperature for 28 h. Then, 1 b (1.05 g, 4.00 mmol), dissolved in CH_2_Cl_2_ (20 ml), is added and stirring is continued for 2 h. The reaction mixture is extracted with three 50 ml portions of pentane. CH_2_Cl_2_ is added when the reaction mixture became too viscous for further extractions. The extracts are filtered and evaporated under reduced pressure. The crude product was purified by column chromatography on silica gel with CH_2_Cl_2_/petroleum ether (1:1). Slow evaporation afforded white crystals of 2 b (Yield: 94%). mp 183°C, ^1^H NMR (CDCl_3_): 3.00 (m, 7H, CH_2_), 3.24 (m, 1H, CH_2_), 6.43 (d,2*H*, *J *= 8.2 Hz, H_aromatic_), 6.59 (d, 2H, *J *= 2.1 Hz, H_aromatic_), 6.65 (dd, 2H, *J *= 8.2 Hz, *J *= 2.1 Hz, H_aromatic_), 7.43(s, 2H, C*H*=CBr_2_) p.p.m.. ^13^C{^1^H}NMR (CDCl_3_): 34.9, 35.4 (C-1, C-2, C-9, C-10), 90.5 (C-18,*C*-20), 130.5, 134.0, 135.7, 135.8 (C-5, C-7, C-8, C-12, C-13, C-15, C-16,*C*-17), 136.2 (C-4, C-16), 138.1, 139.3 (C-3, C-6, C-11, C-14) p.p.m.. UV-vis (CH_2_Cl_2_)[l_max _nm (*e*)]: 229 (57544 *M*^-1^.cm^-1^),266 (20417 *M*^-1^.cm^-1^). Anal. Calcd for C_20_H_16_Br_4_:*C*, 41.71, H, 2.80, Found: C, 41.63, H, 2.71.(Clément *et al.*, 2007*b*).

### Refinement

Refinement was accomplished by full-matrix least-squares methods (based on Fo2, *SHELXL97*); anisotropic thermal parameters for all non-H atoms in the final cycles; hydrogen atoms were placed in calculated positions and refined using a riding model with *U*_iso_(H) = 1.2 *U*_eq_(C).

### Crystal data

**Table:**

C_20_H_16_Br_4_	*D*_x_ = 2.048 Mg m^−^^3^
*M**_r_* = 575.97	Melting point: 456 K
Orthorhombic, *P**b**c**a*	Mo *K*α radiation, λ = 0.71073 Å
Hall symbol: -P 2ac 2ab	Cell parameters from 1766 reflections
*a* = 12.155 (1) Å	θ = 2.2–27.0°
*b* = 8.3819 (9) Å	µ = 8.62 mm^−^^1^
*c* = 18.335 (2) Å	*T* = 173 K
*V* = 1867.9 (3) Å^3^	Irregular, white
*Z* = 4	0.30 × 0.20 × 0.10 mm
*F*(000) = 1104	

### Data collection

**Table:**

Bruker APEX CCD diffractometer	2043 independent reflections
Radiation source: fine-focus sealed tube	1766 reflections with *I* > 2σ(*I*)
graphite	*R*_int_ = 0.045
CCD scans	θ_max_ = 27.0°, θ_min_ = 2.2°
Absorption correction: multi-scan (*SADABS*; Bruker, 1999)	*h* = −15→15
*T*_min_ = 0.141, *T*_max_ = 0.418	*k* = −10→10
16030 measured reflections	*l* = −23→23

### Refinement

**Table:**

Refinement on *F*^2^	Primary atom site location: structure-invariant direct methods
Least-squares matrix: full	Secondary atom site location: difference Fourier map
*R*[*F*^2^ > 2σ(*F*^2^)] = 0.032	Hydrogen site location: inferred from neighbouring sites
*wR*(*F*^2^) = 0.073	H-atom parameters constrained
*S* = 1.08	*w* = 1/[σ^2^(*F*_o_^2^) + (0.0298*P*)^2^ + 3.2824*P*] where *P* = (*F*_o_^2^ + 2*F*_c_^2^)/3
2043 reflections	(Δ/σ)_max_ = 0.001
109 parameters	Δρ_max_ = 0.84 e Å^−^^3^
0 restraints	Δρ_min_ = −0.60 e Å^−^^3^

### Special details

**Table:**

Geometry. All e.s.d.'s (except the e.s.d. in the dihedral angle between two l.s. planes) are estimated using the full covariance matrix. The cell e.s.d.'s are taken into account individually in the estimation of e.s.d.'s in distances, angles and torsion angles; correlations between e.s.d.'s in cell parameters are only used when they are defined by crystal symmetry. An approximate (isotropic) treatment of cell e.s.d.'s is used for estimating e.s.d.'s involving l.s. planes.
Refinement. Refinement of *F*^2^ against ALL reflections. The weighted *R*-factor *wR* and goodness of fit *S* are based on *F*^2^, conventional *R*-factors *R* are based on *F*, with *F* set to zero for negative *F*^2^. The threshold expression of *F*^2^ > σ(*F*^2^) is used only for calculating *R*-factors(gt) *etc*. and is not relevant to the choice of reflections for refinement. *R*-factors based on *F*^2^ are statistically about twice as large as those based on *F*, and *R*- factors based on ALL data will be even larger.

### Fractional atomic coordinates and isotropic or equivalent isotropic displacement parameters (Å^2^)

**Table:**

	*x*	*y*	*z*	*U*_iso_*/*U*_eq_	
Br1	0.94392 (3)	0.12568 (5)	0.72066 (2)	0.03823 (13)	
Br2	1.18942 (3)	0.01277 (4)	0.70021 (2)	0.03403 (12)	
C1	1.0695 (3)	0.1284 (4)	0.66105 (19)	0.0223 (7)	
C2	1.0750 (3)	0.1954 (4)	0.59555 (18)	0.0216 (7)	
H2	1.1423	0.1819	0.5699	0.026*	
C3	0.9893 (3)	0.2882 (4)	0.55781 (18)	0.0198 (6)	
C4	0.9745 (3)	0.2705 (4)	0.48219 (18)	0.0215 (7)	
C5	0.8859 (3)	0.3511 (4)	0.45039 (19)	0.0250 (7)	
H5	0.8626	0.3226	0.4027	0.030*	
C6	0.8316 (3)	0.4717 (4)	0.4873 (2)	0.0241 (7)	
H6	0.7720	0.5255	0.4645	0.029*	
C7	0.8632 (3)	0.5151 (4)	0.55752 (19)	0.0227 (7)	
C8	0.9336 (3)	0.4115 (4)	0.59427 (18)	0.0213 (7)	
H8	0.9442	0.4246	0.6453	0.026*	
C9	0.8418 (3)	0.6819 (4)	0.5856 (2)	0.0280 (8)	
H9A	0.8312	0.6778	0.6391	0.034*	
H9B	0.7729	0.7228	0.5636	0.034*	
C10	1.0610 (3)	0.1987 (4)	0.43258 (19)	0.0263 (7)	
H10A	1.0256	0.1650	0.3864	0.032*	
H10B	1.0918	0.1023	0.4562	0.032*	

### Atomic displacement parameters (Å^2^)

**Table:**

	*U*^11^	*U*^22^	*U*^33^	*U*^12^	*U*^13^	*U*^23^
Br1	0.0365 (2)	0.0460 (2)	0.0321 (2)	0.01147 (17)	0.01440 (17)	0.01208 (17)
Br2	0.0273 (2)	0.0414 (2)	0.0335 (2)	0.00426 (16)	−0.00318 (15)	0.01310 (16)
C1	0.0172 (15)	0.0229 (15)	0.0267 (17)	0.0017 (13)	−0.0003 (13)	−0.0005 (13)
C2	0.0174 (15)	0.0218 (15)	0.0256 (17)	0.0000 (12)	0.0026 (13)	0.0015 (13)
C3	0.0163 (15)	0.0181 (15)	0.0251 (17)	−0.0045 (12)	0.0000 (13)	0.0023 (12)
C4	0.0238 (17)	0.0148 (14)	0.0260 (17)	−0.0054 (12)	−0.0006 (13)	−0.0006 (12)
C5	0.0257 (17)	0.0270 (17)	0.0223 (18)	−0.0090 (14)	−0.0035 (14)	0.0024 (13)
C6	0.0173 (16)	0.0240 (16)	0.0309 (19)	−0.0051 (13)	−0.0015 (14)	0.0065 (14)
C7	0.0182 (16)	0.0245 (16)	0.0254 (18)	−0.0033 (13)	0.0047 (13)	0.0034 (13)
C8	0.0198 (16)	0.0228 (16)	0.0212 (16)	−0.0022 (13)	0.0011 (13)	0.0024 (12)
C9	0.0298 (18)	0.0275 (17)	0.0266 (19)	0.0080 (14)	0.0060 (15)	0.0040 (14)
C10	0.0342 (19)	0.0224 (15)	0.0223 (17)	−0.0003 (14)	−0.0004 (15)	−0.0027 (13)

### Geometric parameters (Å, °)

**Table:**

Br1—C1	1.878 (3)	C6—C7	1.392 (5)
Br2—C1	1.892 (3)	C6—H6	0.9500
C1—C2	1.328 (5)	C7—C8	1.392 (4)
C2—C3	1.473 (4)	C7—C9	1.512 (5)
C2—H2	0.9500	C8—H8	0.9500
C3—C8	1.405 (4)	C9—C10^i^	1.584 (5)
C3—C4	1.406 (5)	C9—H9A	0.9900
C4—C5	1.399 (5)	C9—H9B	0.9900
C4—C10	1.515 (5)	C10—C9^i^	1.584 (5)
C5—C6	1.385 (5)	C10—H10A	0.9900
C5—H5	0.9500	C10—H10B	0.9900
			
C2—C1—Br1	124.9 (2)	C6—C7—C8	117.0 (3)
C2—C1—Br2	121.4 (2)	C6—C7—C9	120.5 (3)
Br1—C1—Br2	113.53 (17)	C8—C7—C9	121.2 (3)
C1—C2—C3	127.8 (3)	C7—C8—C3	121.6 (3)
C1—C2—H2	116.1	C7—C8—H8	119.2
C3—C2—H2	116.1	C3—C8—H8	119.2
C8—C3—C4	119.0 (3)	C7—C9—C10^i^	112.6 (3)
C8—C3—C2	120.4 (3)	C7—C9—H9A	109.1
C4—C3—C2	119.9 (3)	C10^i^—C9—H9A	109.1
C5—C4—C3	117.3 (3)	C7—C9—H9B	109.1
C5—C4—C10	118.4 (3)	C10^i^—C9—H9B	109.1
C3—C4—C10	123.0 (3)	H9A—C9—H9B	107.8
C6—C5—C4	121.1 (3)	C4—C10—C9^i^	113.1 (3)
C6—C5—H5	119.5	C4—C10—H10A	109.0
C4—C5—H5	119.5	C9^i^—C10—H10A	109.0
C5—C6—C7	120.7 (3)	C4—C10—H10B	109.0
C5—C6—H6	119.6	C9^i^—C10—H10B	109.0
C7—C6—H6	119.6	H10A—C10—H10B	107.8

Symmetry codes: (i) −*x*+2, −*y*+1, −*z*+1.
